# Recommendations for the use of mathematical modelling to support decision‐making on integration of non‐communicable diseases into HIV care

**DOI:** 10.1002/jia2.25505

**Published:** 2020-06-19

**Authors:** Joseph Kibachio, Valerian Mwenda, Oren Ombiro, Jamima H Kamano, Pablo N Perez‐Guzman, Kennedy K Mutai, Idris Guessous, David Beran, Paratsu Kasaie, Brian Weir, Blythe Beecroft, Nduku Kilonzo, Linda Kupfer, Mikaela Smit

**Affiliations:** ^1^ Division of Non‐communicable Diseases Ministry of Health Kenya; ^2^ Faculty of Medicine University of Geneva Switzerland Geneva; ^3^ Department of Medicine Moi University School of Medicine Kenya Eldoret; ^4^ AMPATH Kenya London; ^5^ MRC Centre for Global Infectious Disease Analysis Department of Infectious Disease Epidemiology Imperial College London London United Kingdom; ^6^ National AIDS Control Council Kenya London; ^7^ Division of Primary Care Medicine Geneva University Hospital and University of Geneva Geneva Switzerland; ^8^ Division of Tropical and Humanitarian Medicine University of Geneva and Geneva University Hospitals Geneva Switzerland; ^9^ John Hopkins Bloomberg School of Public Health Baltimore MD USA; ^10^ Fogarty International Center National Institutes of Health Bethesda MD USA

**Keywords:** policy, integration, modelling, Kenya, non‐communicable diseases, HIV

## Abstract

**Introduction:**

Integrating services for non‐communicable diseases (NCDs) into existing primary care platforms such as HIV programmes has been recommended as a way of strengthening health systems, reducing redundancies and leveraging existing systems to rapidly scale‐up underdeveloped programmes. Mathematical modelling provides a powerful tool to address questions around priorities, optimization and implementation of such programmes. In this study, we examine the case for NCD‐HIV integration, use Kenya as a case‐study to highlight how modelling has supported wider policy formulation and decision‐making in healthcare and to collate stakeholders’ recommendations on use of models for NCD‐HIV integration decision‐making.

**Discussion:**

Across Africa, NCDs are increasingly posing challenges for health systems, which historically focused on the care of acute and infectious conditions. Pilot programmes using integrated care services have generated advantages for both provider and user, been cost‐effective, practical and achieve rapid coverage scale‐up. The shared chronic nature of NCDs and HIV means that many operational approaches and infrastructure developed for HIV programmes apply to NCDs, suggesting this to be a cost‐effective and sustainable policy option for countries with large HIV programmes and small, un‐resourced NCD programmes. However, the vertical nature of current disease programmes, policy financing and operations operate as barriers to NCD‐HIV integration. Modelling has successfully been used to inform health decision‐making across a number of disease areas and in a number of ways. Examples from Kenya include (i) estimating current and future disease burden to set priorities for public health interventions, (ii) forecasting the requisite investments by government, (iii) comparing the impact of different integration approaches, (iv) performing cost‐benefit analysis for integration and (v) evaluating health system capacity needs.

**Conclusions:**

Modelling can and should play an integral part in the decision‐making processes for health in general and NCD‐HIV integration specifically. It is especially useful where little data is available. The successful use of modelling to inform decision‐making will depend on several factors including policy makers’ comfort with and understanding of models and their uncertainties, modellers understanding of national priorities, funding opportunities and building local modelling capacity to ensure sustainability.

## 1 Introduction

The growing burden of non‐communicable diseases (NCDs) in low‐ and middle‐income countries calls for concerted efforts at prevention, early detection and optimization of health systems for effective chronic care delivery. Given the multi‐morbid nature of NCDs [1, 2], it also calls for a shift from fragmented health systems to more integrated and holistic care provision [3].

One of the approaches policy makers in countries with poorly resourced NCD programmes could consider is integration of chronic care services into existing robust primary health structures. An example of where this is taking place is Kenya, whose National Strategy for Prevention and Control of NCDs 2015 to 2020 emphasizes linkage of care between major NCDs and communicable diseases such as human immunodeficiency virus/acquired immune deficiency syndrome (HIV/AIDS) and tuberculosis (TB) [4]. Separate care models can result in redundancies at the system, service and patient level, such as separate training programmes, laboratory infrastructure and data systems [5, 6]. Integration is premised on the assumption that these redundant edges in well financed primary care platforms can be leveraged for under‐resourced and under‐developed programmes such as those for NCDs and that there exists potential for synergies and shared benefits for both provider and user in delivering integrated and comprehensive care packages.

However, many challenges and barriers to implementation of integrated service provision remain which necessitate evidence‐based research to facilitate the translation of strategic and policy commitments to practical changes on the ground. Mathematical models have provided evidence‐based guidance for decision‐making around priorities, optimization and implementation of services. Although no modelling study has focused on the systematic evaluation of integration of NCD services into existing platforms, there are many examples from Kenya and the wider region of how mathematical models have supported decision‐making more generally.

In this study, we examine the case for NCD‐HIV integration, use Kenya as a case‐study to highlight how modelling has supported wider policy formulation and decision‐making in healthcare and to collate stakeholders’ recommendations on use of models for NCD‐HIV integration decision‐making.

## 2 Discussion

### 2.1 The burden of NCDs in sub‐Saharan Africa

Across sub‐Saharan Africa (SSA), NCDs are the second leading cause of morbidity and mortality after HIV/AIDS [7], yet global financing for NCDs comprises less than 2% of total health expenditure [8]. Studies from both high income countries and LMICs have shown that people living with HIV (PLHIV) experience a higher NCD burden [2, 9, 10]. A recent modelling study estimates that 51% of Kenyan adults currently suffer from ≥1 NCD, that this burden was higher in PLHIV compared to HIV negative and is projected to increase [11]. It identified hypertension, elevated total cholesterol, diabetes, chronic kidney disease and depression as the most prevalent NCDs, with cardiovascular disease and cancer as the main NCD‐related causes of deaths, irrespective of HIV status [11]. While the mechanisms of NCDs in the context of HIV are not fully understood, they likely involve complex interactions between traditional risk factors, including smoking, diet, and exercise, and HIV‐specific risk factors, including long‐term immune activation, inflammation and toxicity related to long‐term ART use [2].

Every country in the region will have outlined their priorities for NCDs in their national strategic plan. In Kenya, the National Strategy for Prevention and Control of NCDs 2015 to 2020 lays emphasis on four major NCDs: cardiovascular conditions, cancers, diabetes, and chronic obstructive pulmonary diseases and their shared risk factors [4]. The Kenyan Poverty Commission found that NCDs decrease household income by an estimated 29% and can subject families to catastrophic expenditures and poverty [12]. This threatens the achievement of Universal Health Coverage (UHC) aspired by the region, as one of the pillars of UHC is to cushion individuals, households and communities from catastrophic and impoverishing health expenditures [13].

### 2.2 The case for integrated care

Integration of health services is the foundation of primary healthcare and will form the foundation of UHC [14]. Integration has been shown to generate advantages for both provider and user, and has been demonstrated to be cost‐effective, practical and rapidly scalable [15, 16, 17]. For the users, integration can increase equity, decrease stigma associated with healthcare demand, improve access to services and disease outcomes [18]. For example, The Integrated Management of Childhood Illness initiative uses a comprehensive primary care‐based service delivery model to reduce both morbidity and mortality and promote improved health childhood development [19]. From the supply side, integration can generate economies of scope and reduce redundancies in resource limited settings [14]. For example, leveraging existing infrastructure such as buildings, laboratory and supply chains can generate economic savings while joint supervision, training and mentorship has been shown to reduce demand on health workers' time [14].

### 2.3 Forms of integration

Integration may take various forms [14, 20], with many approaches already successfully operating in SSA. In Kenya, integration to date is mainly in the areas of infectious disease and maternal and child health. Integration can focus on providing a package of preventive and curative health interventions for a particular population group, such as the “Integrated Management of Childhood Illnesses” programme. Similarly, integration can involve offering multiple services for diseases requiring common interventions under “one roof,” such as integrating nutritional services in Diabetes Centers of Excellence which include integration of laboratory and supply chains. Finally, integration at the policy level can include jointly agreed health sector strategies, joint health sector performance reviews and sector‐wide approaches.

### 2.4 HIV as an example of integrated care

The HIV response provides, perhaps, the best example of how integration can be successfully operationalized for chronic conditions. Despite being an infectious disease, care for HIV has evolved into a chronic care model, that involves patient follow‐up, continuity of care, monitoring and auxiliary services to maintain patients’ health and quality of life. HIV/AIDS prevention and treatment services have been successfully integrated with services focused on maternal and child health, TB, nutritional advice, family planning services, lifestyle advice services and screening programmes for NCDs [21, 22, 23], and has established strong health systems, financing and infrastructure across many LMIC settings.

### 2.5 The case for NCD‐HIV integration

It is clear, given the large and growing burden of NCDs in both PLHIV and the general population across SSA, that services for the screening and treatment of NCDs will play an important role in the preservation of health. Building on HIV platforms could shorten the learning curve for NCD prevention and control [24, 25, 26, 27], particularly for countries with large HIV programmes and small, un‐resourced NCD programmes. Considering their shared chronic nature, a majority of the programmatic and operational approaches and infrastructure developed for HIV programmes could be used for NCDs, especially in resource‐constrained settings [18]. For instance, the surveillance systems that have been used in the HIV response can be leveraged to quantify the magnitude of NCDs, the cost of prevention and management, identify vulnerable population groups and assess the effects of policy and operational interventions [24]. Other potential areas of integration for NCDs include peer support, m‐Health and community‐based screening [17]. In fact in Uganda leveraging the HIV prevention and care infrastructure to deliver multi‐disease services (hypertension and diabetes) resulted in marginal incremental cost of integrating screening for these NCDs compared with the cost of HIV testing [28].

Despite the numerous merits of NCD‐HIV integration, concerns remain, including that integration may compromise existing successes and reverse HIV advances that have been achieved. There are concerns around (i) inequity in NCD care provision in early phases of implementation, with more NCD care for PLHIV than the general population, (ii) how service provision designed for low‐prevalence diseases could be scaled up rapidly enough to deal with highly prevalence NCDs such as hypertension and (iii) how individual and environmental barriers to NCD care seeking behaviour can be overcome [29]. Other challenges to providing fully funded programmes at no or low cost to patients include the need for significant upfront investments, provider training and set up of robust supply chains. This is further compounded by the exclusivity that characterizes current vertical disease programming, policy, financing and operations. Finally, NCDs are complex and attract low financing, while an expectation of free services and medications was created by HIV care.

In this era of UHC and with the push towards more domestic financing, the potential benefits seem to outweigh the risks of integration, however, by providing opportunities to strengthen the health system at large. Nevertheless, each disease entity within NCDs has its unique challenges, and these should be considered when planning for integration. As Kenya and other countries around the region focus on rolling‐out integrated NCD‐HIV programmes, they will need to be guided by robust evidence around priorities, optimization and implementation of these programmes in order to both ensure return on investment and safe‐guarding of existing programmes.

### 2.6 Why mathematical modelling?

Mathematical modelling provides a powerful synthesizing tool, with multiple applications in the health sector and policy development. Although to date no modelling study has focused on systematic evaluation of integration of NCD services into existing platforms, there are many examples of how mathematical models have supported decision‐making, particularly in the field of infectious diseases and HIV. In this section, we use Kenya as a case‐study to highlight how modelling has supported wider policy formulation and decision‐making in healthcare and later collate stakeholders’ recommendations on use of models for NCD‐HIV integration decision‐making. While we focus on Kenya as a case study, the lessons, priorities and recommendations identified will apply to other LMICs with large HIV and un‐resources NCD programmes and to the use of modelling in decision‐making more widely.

### 2.7 The role of mathematical models in estimating disease burden

Estimates of disease burden, as well as projections of how these may change over time are crucial to inform strategic planning of health services in the country, yet surveillance systems in many LMIC countries still focus on capturing data on only a handful of key areas, such as infectious diseases, child and maternal health and death registries. Accurate NCD data for policy utility has been a major bottleneck in all SSA due to the lack of surveillance systems for these diseases. Data on NCDs in many countries, including Kenya largely derived from the WHO Stepwise Survey [30] or geographically limited, usually pilot, research studies. Kenya is in the process of strengthening NCD indicators in the national health information systems to provide routine reliable data to inform planning. To bridge the current data gap, mathematical modelling utilizing multiple data sources to extrapolate NCD outcomes provides an opportunity to improve the availability and accuracy of locally relevant data for policy and programming.

There are many examples of how mathematical models have been used to establish the burden of individual infectious diseases and generate risk maps, for example HIV, TB and malaria at national or sub‐national levels across SSA [31, 32, 33, 34] and have long been used to generate annual HIV estimates that aid in planning and resource mobilization in Kenya. However, few models have establish the burden of multimorbidity of NCDs [11, 35, 36, 37, 38]. In 2019, modelling was used to provide the first‐ever national estimates of six NCDs and eight cancers by HIV status in Kenya, by combining a data landscaping exercise of available NCD data, and triangulating it with demographic data in a modelling framework [11]. The results will be summarized in the first ever national report on NCD estimates in 2020 and will help inform priorities around integrated NCD‐HIV activities.

### 2.8 The role of modelling in optimizing healthcare provision

Within the realms of health system optimization, models have been used to identify health care priorities, including systematic comparison of prevention measures, and evaluations of the cost‐effectiveness of integrating health services [39, 40, 41, 42, 43]. Many of these findings have fed directly into national and global policy. For example, the 2014/2015 to 2018/2019 Kenya AIDS Strategic framework includes recommendations informed by a modelling exercise [40, 44]. This model analysis found that selectively targeting primary HIV prevention interventions to population and regions at highest risk of HIV could achieve a 55% reduction in new HIV cases by 2030, compared to 40% when interventions were adopted uniformly across the country [40]. More recently, the World Health Organization launched its global strategy for cervical cancer elimination, which was informed by an extensive modelling consultation [41].

### 2.9 The role of mathematical modelling in exploring health system capacity needs

Finally, models have also been used to explore questions around task‐shifting, human resources needs, and optimization of health financing mechanisms [45, 46, 47, 48]. In Kenya, one study looked at long‐term economic impact of return on investment and found that shifting cognitive behavioural therapy to reduce alcohol abuse among PLHIV to paraprofessionals is effective and economical and averts alcohol‐related morbidity and mortality [45]. Another study evaluated optimal financial mechanisms to sustain UHC in Kenya, including social health insurance and general tax‐funding mechanisms [46]. The study provided recommendations for long‐term financial sustainability, which included a tax‐funding system and innovative financing options [46].

### 2.10 Recommendations for the use of mathematical modelling to support NCD‐HIV integration

NCD‐HIV integration appears to be a cost‐effective and sustainable policy option for countries with large HIV programmes and small, un‐resourced NCD programmes to rapidly scale‐up their NCD programmes, and has been fully adopted by the Kenya’s National Strategy for Prevention and Control of NCDs 2015 to 2020 [4]. Yet several key policy level research gaps for NCD‐HIV integration remain to be addressed, to ensure these programmes are successful. Modelling has proven to be a powerful tool to support decision‐making. We carried out stakeholder consultation and collation of targeted expert opinions. This was done in a snow balling activity between June and September 2019, and included modellers from several international institutions who have supported evidence generation for policy, funders who have worked on the interface between research and policy and policy makers in Kenya from across the Divisions of Cancers, NCDs, HIV and the strategic team at the Ministry of Health. The focus of this consultation was to (i) define key questions around NCD‐HIV integration, (ii) identify where and how to integrate modelling within the policy making process, (iii) identify pre‐requisites for the successful use of models in policy formulation and decision‐making.

**Figure 1:**
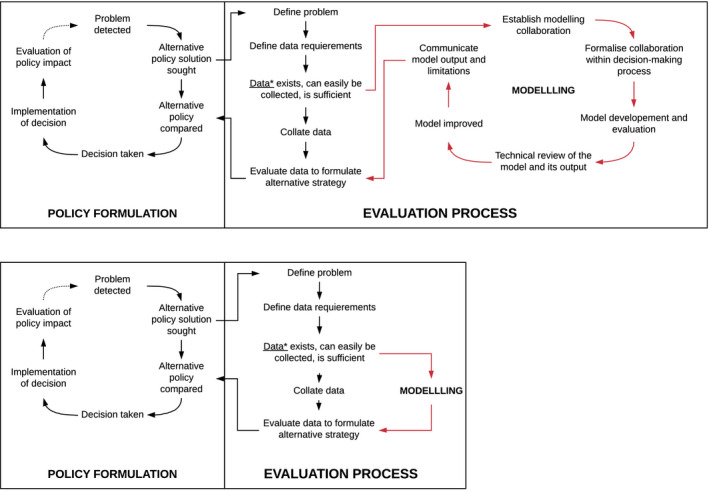
The role of mathematical modelling to inform policy decisions on integrated care for multi‐morbidity in Kenya. *Data referring to primary or programmatic data and expert opinions.

The consultation highlighted eight key priority research question questions from national stakeholders, which can be addressed by modelling (Table 1). Within the well‐defined steps of policy and decision making for health, we suggest that modelling methodology is likely to provide a critical entry point for enhancing these integration efforts in various ways. Policy formulation is driven by the need to provide alternative strategies or guidance for a given gap in health provision and is supported by a formal evaluation process (Figure 1). Modelling can be an important tool in the evaluation process, particularly in areas were little data exist or data collection is weak or unfeasible (Figure 1). The consultation highlighted that the use of modelling for policy formulation and decision making should be accompanied by defined processes, including formal integration into the decision‐making process, robust technical review and dissemination (Figure 1 and Table 1).

**Table 1:** Summary of priority research questions on the pathway to integration as collated through consultation with key stakeholders

What is the impact of integration on improving access to primary prevention services? What is the optimal entry‐points for integration (e.g. HIV platforms, child health to deliver health services to siblings and mothers)? What risk does integration pose at jeopardizing the gains made in the primary platform, for example, HIV programme? Are there economies of scope relating to integration of individual services? How does regional disease prevalence affect the cost‐effectiveness of integration? What is the impact of reducing or removing user fees on cost‐effectiveness of integration/what are the optimal user fees contribute for services under UHC? Within which laboratory sample transport system should NCD diagnostic samples be integrated? What components have the greatest impact when integrated along the continuum of care and what are the markers of success?

HIV, human immunodeficiency virus; NCDs, Non‐communicable diseases; UHC, Universal Health Coverage.

Several prerequisites are were identified through the consultation, for modelling to drive the integration agenda in a sustainable manner (Table 2). First, it emphasized that in order to successfully use modelling to support decision‐making, the application of the models will need to be aligned with the current national aspiration and their use will need to gain wider acceptance as well as the backing of policy makers. Second, policymakers need to be sensitized on the role of modelling in public health, its approaches and techniques, assumptions and limitations. A strong and honest collaboration between modellers and policy makers is crucial to harness the potential for modelling in enhancing the integration agenda. Third, results from models should be widely disseminated, processes evaluated and validated. Models should, as with laboratory experiments, be sufficiently transparent that their results can be replicated. Fourth, models should be linked to existing surveillance and national health information systems, to ensure models serve a complementing, not a duplicating or replacing function. A case example in Kenya is in HIV surveillance system, whereby routine reporting and periodic surveys is combined with modelling to provide up‐to‐date information continuously.

**Table 2:** Key stakeholder recommendations to formally and sustainably integrate modelling in policy formulation and decision‐making

Align modelling with current national priorities Sensitize policy makers to the role of modelling in policy formulation and decision‐making Ensure wider acceptance as well as the backing of policy makers for modelling Develop a set of guidelines to evaluate the transparency, robustness and replicability of models Develop a formal review of model design and output by a national technical team trained in modelling Disseminate results from any policy/modelling exercise and highlight the model’s limitations Link models to the formal national health information systems to avoid duplication and increase efficiencies Foster collaboration with established institutions that routinely utilize models to ensure knowledge transfer Incorporate modelling in public health training in local institutions to build modelling capacity Identify national resources to support sustainability and institutionalization of mathematical modelling

Finally, application of modelling in public health planning and policy formulation must be conducted in a sustainable manner and include human resource capacity for modelling. Several approaches can be utilized for this purpose: availing of resources to institutionalize, maintain and sustain mathematical models to enhance visibility on their role, foster collaboration among institutions that routinely utilize modelling to enhance partnerships and knowledge transfer and incorporating modelling in local public health training to increase the skill pool and create a critical mass of modellers. A critical bottleneck remains the sustainability of these efforts. Modelling to inform policy frequently involves collaborations between academic institutions, which generally house modelling capacity, and governmental organizations. Academic groups rely on funding through outside sources, with funding schemes often being project specific and time limited. Altogether this means that collaborations between modellers and governments can suffer from a lack of sustainable funding. Additional funding focused on support for capacity building of in‐country modellers and support for the transfer of models to countries will help ensure sustainability and continuity of efforts.

## 3 Conclusions

It seems clear that mathematical modelling can and should play a central role in future policy formulation and decision‐making as the sub‐Saharan region grapples with questions of integration and focuses on rolling out UHC, particularly given the often limited evidenced‐based data to support decisions. Models have played a central role in informing policy in other disease areas, demonstrating that they can provide a strong platform of credible research. They will undoubtably be able to generate valuable and robust evidence to answer some key questions that remain regarding NCD‐HIV integration in the region (Table 1).

First, by estimating burden, modelling can support decision‐makers in setting priorities for public health policy interventions. This is key for health conditions with inadequate or weak surveillance systems and therefore little data for decision‐making, of which NCDs are a good example. Second, if policy formulation or revision is required, modelling can be utilized for the formulation of optimized options for an integration approach, cost‐benefit analysis for integration as well as evaluating the impact of integration of services. Finally, models can be utilized in conducting projections of future trends of various health conditions and aid in forecasting the requisite government investments to address them effectively. This is particularly vital for SSA as the triple burden of disease phenomenon manifests in the setting of dwindling donor support. For instance, with integration, there is likely to be increased workload for human resources, and the need for additional equipment and commodities and/or. Thorough forecasting will forestall shortages of commodities and/or waste of resources as the models may provide indicative trends.

Integration of health services will require a policy backing for wide acceptability and sustainability beyond specific programmes. In addition, the change in the system of service delivery towards integration will require the interplay of political, technical and administrative action at several levels, including sustained commitment from the government, and the bridging of critical knowledge gaps. Within the well‐defined steps of policymaking for health, we suggest that modelling methodology is likely to provide a critical entry point for enhancing these integration efforts (Figure 1).

Both integration and modelling ought to be aligned with the current national health priorities to gain wide acceptance and backing. A good example is putting all these efforts in the context of expansion of primary healthcare and national rollout of UHC. While encouraging the use of mathematical models, it is critical to emphasize that they are not a replacement of empirical data collection but rather a tool to assist in interpretation of this data to a more useful form.

Successful use of modelling to inform policy and decision‐making will depend on several factors (Table 2) including policy makers’ comfort with and understanding of models and their uncertainties, modellers understanding the policy questions, funding opportunities and building local modelling capacity to ensure sustainability. While we focus on Kenya and NCD‐HIV integration as a case in point, recommendations also apply to other settings and the use of modelling in policy formulation and decision‐making more widely.

It is clear that modelling has played a valuable role in the formulation of policy recommendations and decision‐making across a number of disease areas and in a number of settings to date. As the paucity of NCD data for policy use in Kenya and the wider region continues to hamper policy decisions on integration, mathematical modelling should play an integral part in bridging this gap now and in the future. This paper outlines a set of clear recommendations on how to sustainability integrate modelling into decision making.

## Competing interests

All authors report no conflicts of interest.

## Authors’ Contributions

MS, JK and LK conceived the paper, formulated the overall aim, scope and lens of the manuscript, with all authors contributing to finalizing its outline and scope. MS, VM, JK, OO and PNPG wrote the first draft of the manuscript. MS, JK, VM, OO, BB, BW, LK and PKS led the design and development of all infographics. JK, VM, OO, JHK, MKK and NK led all aspects of the policy landscape and research gaps in Kenya. MS, PNPG, BW and PKS led all model‐related aspects of the manuscript. All authors contributed to the re‐drafting of the manuscript and in the process of approving the final draft.

## Abbreviations

AIDS, Acquired Immune Deficiency Syndrome; HIV, Human Immunodeficiency virus; NCDs, Non‐communicable diseases; PLHIV, People Living with HIV; SSA, Sub‐Saharan Africa; TB, tuberculosis; UHC, Universal Health Coverage.
